# Partitioning the population attributable fraction for a sequential chain of effects

**DOI:** 10.1186/1742-5573-5-5

**Published:** 2008-10-02

**Authors:** Craig A Mason, Shihfen Tu

**Affiliations:** 1 College of Education and Human Development, University of Maine, and Maine's University Center for Excellence in Developmental Disabilities, University of Maine, Orono, ME, USA; 2 5717 Corbett Hall, Room 3, University of Maine, Orono, ME 04469, USA

## Abstract

**Background:**

While the population attributable fraction (PAF) provides potentially valuable information regarding the community-level effect of risk factors, significant limitations exist with current strategies for estimating a PAF in multiple risk factor models. These strategies can result in paradoxical or ambiguous measures of effect, or require unrealistic assumptions regarding variables in the model. A method is proposed in which an overall or total PAF across multiple risk factors is partitioned into components based upon a sequential ordering of effects. This method is applied to several hypothetical data sets in order to demonstrate its application and interpretation in diverse analytic situations.

**Results:**

The proposed method is demonstrated to provide clear and interpretable measures of effect, even when risk factors are related/correlated and/or when risk factors interact. Furthermore, this strategy not only addresses, but also quantifies issues raised by other researchers who have noted the potential impact of population-shifts on population-level effects in multiple risk factor models.

**Conclusion:**

Combined with simple, unadjusted PAF estimates and an aggregate PAF based on all risk factors under consideration, the sequentially partitioned PAF provides valuable additional information regarding the *process* through which population rates of a disorder may be impacted. In addition, the approach can also be used to statistically control for confounding by other variables, while avoiding the potential pitfalls of attempting to separately differentiate direct and indirect effects.

## Background

**Figure 1:**
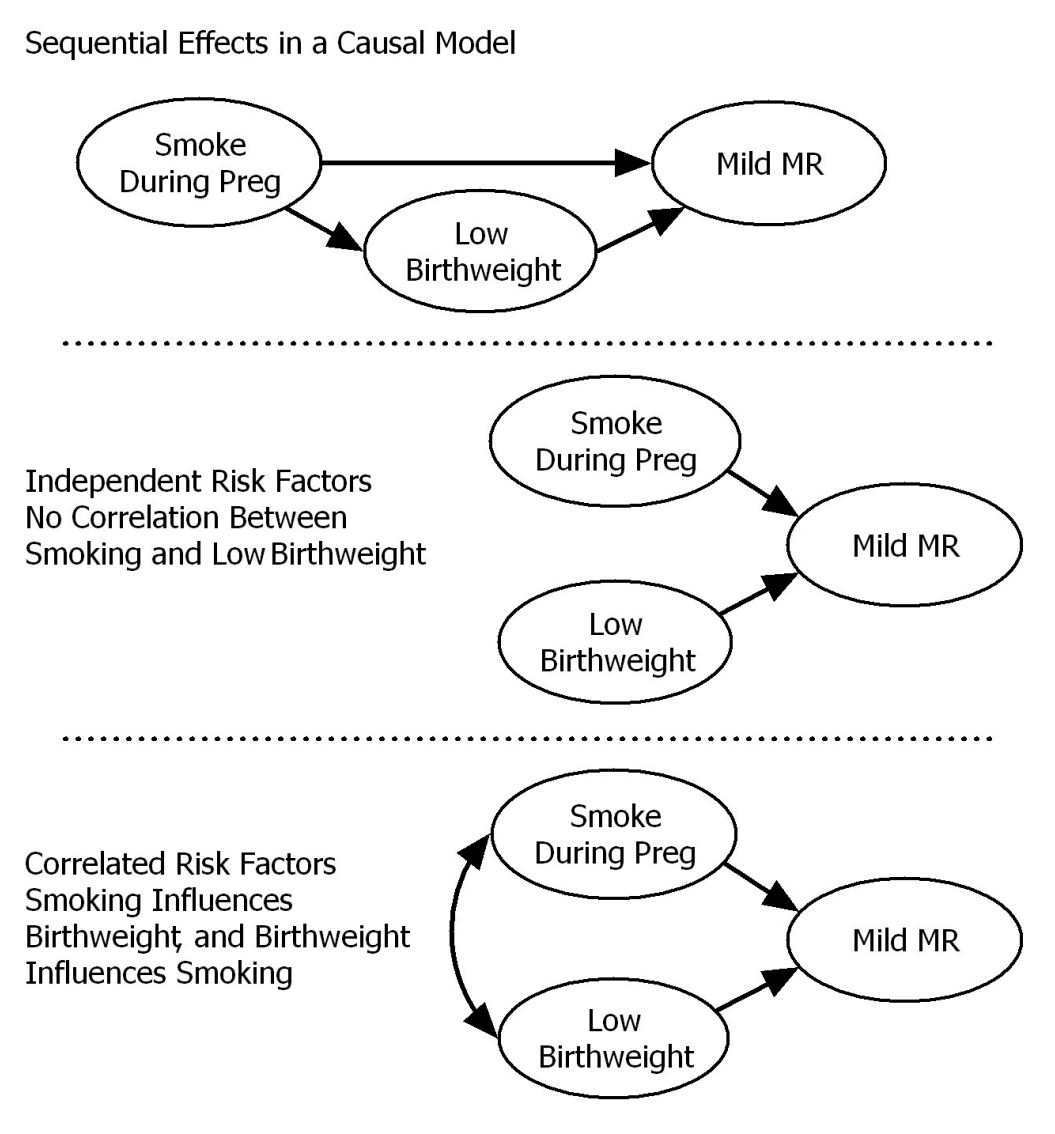
Different Relationships Among Multiple Risk Factors.

Recent attention has focused upon the need to consider the sequential chain of effects when calculating and interpreting relative risk in multiple risk factor models[1]. For example, as illustrated in Figure 1, simultaneously controlling for the mutual association between smoking and birthweight when examining the effect of these variables upon mild mental retardation (MMR) (Figure 1, middle and lower panels) is not equivalent to a model in which smoking leads to elevated risk for low birthweight, which then leads to elevated risk for MMR[2] (Figure 1, top panel). With such models, the manner and sequence in which relative risk is calculated vary depending on the order of the variable in the sequence of effects. A similar issue applies to the estimation of measures of community level effect, such as the population attributable fraction (PAF)–also referred to as population attributable risk, or attributable risk. Ignoring the causal or sequential ordering of risk factors either assumes that they are independent (i.e., do not influence each other–Figure 1, middle panel) or assumes that they are all mutually correlated–every risk factor influences or has bidirectional associations with every other risk factor (Figure 1, bottom panel), even if one occurs in childhood and the other before a child was born.

In a sequential or causal ordering of effects, an earlier risk factor can impact subsequent risk factors by increasing their rate or prevalence (i.e., an indirect effect). In other words, an indirect effect is where one predictor variable has an impact on an outcome variable through an intermediate predictor variable (e.g., smoking influences low birthweight, low birthweight influences MMR–see Figure 1, top panel). In addition, one risk factor may interact with a subsequent risk factor by magnifying or reducing the effect it has upon the outcome (i.e., an interaction effect).

It's worth noting that two predictors can have an indirect effect on an outcome with no interaction effect: For example, smoking may lead to higher rates of low birthweight, and low birthweight may lead to higher rates of MMR; but the effect of being born low birthweight may be identical for all children, regardless of whether or not their mother smoked during pregnancy. Similarly, absence of an indirect effect does not preclude an interaction effect upon the same outcome. For example, child sex and birthweight may have no correlation with each other–and hence no indirect effect–while the effect of low birthweight on a developmental outcome may be very small for females but very large for males (i.e., a large interaction effect).

While several strategies exist for estimating a PAF for one risk factor while simultaneously statistically controlling for other variables [3-5], these strategies do not consider the sequence in which these variables influence each other and the outcome as just described. This results in estimates that have a variety of known problems, including values that are paradoxical, counter-intuitive, or simply nonsensical[6]. These and similar problems have led some to question whether adjusted PAFs are of any practical value [7-10]. Furthermore, these strategies generally involve either estimating the direct effect (e.g., effect of smoking on MMR that is unrelated to birthweight) or the indirect effect (e.g., effect of smoking on MMR that is related to smoking's effect on birthweight–see Figure 1, top panel). However, others have noted various issues with differentiating direct and indirect effects in biological models [7-10], again, raising questions as to the practicality of calculating adjusted PAFs in multiple risk factor models.

In contrast, this paper outlines a procedure for partitioning the overall PAF associated with a group of risk factors into the individual effects associated with each specific risk factor based upon the order of that risk factor in the sequence of effects. As will be described in more detail, this technique directly parallels the estimation of R^2 ^and change in R^2 ^one estimates through a hierarchical multiple regression in which variables are entered in multiple steps, with those that occur earlier in a process (e.g., prenatal factors) entered prior to those that occur later in a process (e.g., early childhood environment). This results in parameter estimates at any given step being adjusted for the effects of those variables that were entered in earlier steps. This same process can be used to adjust for confounding by other variables, such as sex or SES, which may be related to the risk factors and outcome of interest.

It is also worth noting that an additional strength of this approach is that it adjusts a PAF for previously entered effects without attempting to differentiate direct and/or indirect effects. Instead, in estimates the total or net effect of a variable–direct and indirect effects combined–after controlling for other risk factors and/or confounding by other variables.

The proposed procedure is appropriate for representative or population-based studies where estimates of the risk ratio (RR) and the prevalence of a risk factor (p_e_) can be directly estimated. We first briefly describe existing strategies for assessing adjusted PAFs in multiple risk factor models, and then describe the proposed strategy for partitioning a PAF based upon the order of effects, drawing the parallels between this approach and the estimate of R^2 ^and change in R^2 ^in a multiple regression analysis. We illustrate this technique in three scenarios: (1) two risk factors are related/correlated with each other, but do not interact (i.e., there is not interaction effect), (2) two risk factors are not related/correlated with each other, but do interact (i.e., there is an interaction effect), (3) two risk factors are related/correlated with each other and interact.

### Estimation of PAF in stratified models

**Figure 2:**
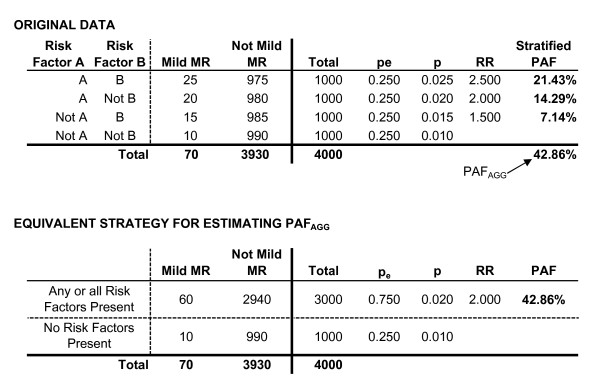
Calculating PAF_AGG_.

The most transparent approach for estimating a PAF across multiple risk factors is to use a stratified model. In a stratified model, the sample is stratified based upon the possible combinations of risk factors, and a PAF is estimated for each combination. The referent group is those without any of the risk factors under consideration. An example of this approach is presented in Figure 2, where risk factor A and risk factor B are risk factors for MMR in children. The referent group consists of children with neither risk factor, and there are three "at-risk" groups: those with A only, those with B only, and those with both A and B. A PAF is calculated for any one these combinations of A and/or B using Equation 1.

(1) $$PAF_{i}=\frac{P_{ei}\left(RR_{i}-1\right)}{1+P_{e1}\left(RR_{1}-1\right)+P_{e2}\left(RR_{2}-1\right)+P_{e3}\left(RR_{3}-1\right)}$$

where *i *indicates which of the three at-risk groups is being estimated, PAF_i _indicates the estimated PAF for the corresponding group, and P_ei _is the proportion of the sample in group *i*. In addition, P_e1_, P_e2_, and P_e3 _indicate the proportion of the sample in each of the three at-risk groups, and RR_1_, RR_2_, and RR_3 _indicate their corresponding risk ratio. In other words, three PAFs are calculated, corresponding to those with A only, B only, and those with both A and B. When calculating these estimates, the denominator does not change; however, the numerator for any given estimate is equal to the proportion of the sample in that risk-group (P_ei_), multiplied by the corresponding risk-ratio minus 1.

There are several limitations with this strategy. Specifically, stratification does not incorporate any sequence of effect between the risk factors, and it assumes that there is no association between the risk factors. However, it should be noted that the sum of the stratified PAFs (PAF_AGG_) is a legitimate estimate of the combined aggregate effect of both risk factors relative to those without either risk factor. In other words, PAF_AGG _estimates the percentage of cases in the population that are associated with either or both A and B regardless of whether A and B are unrelated or whether they are strongly related. This is equivalent to removing any distinction between the two risk factors and simply performing a risk/no risk comparison, as illustrated at the bottom of Figure 2. The issue becomes problematic when one wishes to use stratification to examine the effect specific to either A or B.

### Additional existing strategies for estimating adjusted pafs

In situations where risk factors are related, several formulas exist for estimating adjusted PAF's [3-5]. These techniques involve adjusting the relative risk for one variable for the effect of other variables, and then using this adjusted relative risk for estimating a PAF. For example, using a Mantel-Haenszel odds ratio, an adjusted PAF can be estimated using Equation Two...

(2) $$PAF^{\prime }=p_{e}\frac{OR_{MH}-1}{OR_{MH}}$$

where p_e _is estimated as the ratio of the number of exposed cases, relative to the total number of cases, and OR_MH _is the Mantel-Haenszel odds ratio. The result is a PAF adjusted for the effect of other risk factors in the model. Alternatively, others have proposed strategies for estimating an adjusted PAF through a multiple logistic regression[3]. In general, these approaches use logistic regression to calculate an odds-ratio adjusted for other effects, and then uses this adjusted odds-ratio to estimate a PAF.

While resolving the issue of related/correlated risk factors, these approaches have their own limitations. As noted by Rowe and colleagues, individual, unadjusted PAFs can sum to more than 1.0 because a person with more than one risk factor can have a disorder prevented (or caused) in more than one way[6]. For example, if the combination of two risk factors is a sufficient cause for a disorder, cases among individuals with both risk factors will be "double counted" when calculating a PAF for each of these risk factors. Consequently, one might expect adjustment techniques to remove this "overlapping" risk [11] and result in adjusted PAFs that do not sum to more than 1.0.

However, as demonstrated by Coughlin and colleagues[12], many of these techniques fail in this regard. Specifically, Coughlin and colleagues examined birthweight and maternal consumption of processed lunchmeat as risk factors for childhood astrocytoma. Using a logistic model, the authors found that the PAF for birthweight and processed meat considered jointly was equal to .791. After adjusting each risk factor for its association with the other, the authors reported that the adjusted PAF for birthweight was equal to .558, while the adjusted PAF for processed lunchmeat was .521. Not only did the adjusted PAFs sum to more than the joint PAF when both variables were aggregated, the adjusted PAFs summed to more than 1.0. In this same paper, Coughlin and colleagues propose an adjustment strategy in which the adjusted PAFs will sum to the joint, aggregate PAF for both risk factors together [12]; however, this strategy, as well as other techniques for calculating adjusted PAFs, does not address the sequence of effects. Instead, they simultaneously remove the effect of all other variables upon each other without considering how earlier risk factors may impact the prevalence of later risk factors.

### An alternative strategy: partitioning a paf sequentially

#### Background

The method being proposed here is based on an alternative approach to estimating adjusted PAFs. As described below, the method can be seen as being somewhat analogous to partitioning R^2 ^in a multi-step, hierarchical multiple regression, where the estimation of r^2 ^(i.e., the net or total effect of a single variable), total R^2 ^(i.e., the net or total effect of a set of variables), and change in R^2 ^(i.e., the net effect of a variable(s) after controlling for previously entered variables) in a multiple regression analysis[13,14] have parallels in a simple/unadjusted PAF, an aggregate PAF, and an adjusted PAF. This point is illustrated through a hypothetical study using multiple regression, in which child sex, early childhood parenting, and adolescent peer behavior serve as predictors of adolescent problem behavior.

##### Simple Effects

One might begin such a study by examining the individual simple r^2 ^of each of these three predictors in relation to adolescent problem behavior. In our hypothetical example, this might result in r^2 ^= .30 for child sex, r^2 ^= .35 for early childhood parenting, and r^2 ^= .40 for adolescent peer behavior. There is nothing inherently wrong with these three r^2 ^estimates–each describes the total or net association between the corresponding predictor and the outcome (adolescent problem behavior).

This has a direct parallel with PAF estimates in multiple risk factor models. Consider a simple alternative example, where maternal smoking during pregnancy and low birth weight are predictors of MMR in elementary school. One can estimate a simple, unadjusted PAF for smoking and a simple, unadjusted PAF for birth weight–and these would be entirely valid estimates of the total or net association between these risk factors and MMR.

##### Aggregate/Total Effects

Returning to the multiple regression example, if one was interested in simultaneously examining the *total *effect of all three predictors combined, simply adding the unadjusted r^2^'s would result in a sum of 1.05, an impossible and nonsensical solution. This reflects the lack of independence among the predictors (i.e., the predictors are related to each other), with some of the effect being shared across these variables. Instead, one could perform a multiple regression by entering all three predictors in a single step. In this hypothetical example, we will assume that this results in an R^2 ^of .45, indicating that the three variables as a group account for 45% of the variance in adolescent problem behavior scores.

Again, this has a direct parallel with PAF estimates in multiple risk factor models. If one was interested in simultaneously examining the effect of multiple predictors and simply added their unadjusted PAFs, the result would not only be invalid, but could be impossible or nonsensical. Instead, one can estimate a total or aggregate PAF (PAF_AGG_) by comparing those with none of the risk factors of interest to those with one or more of the risk factors. Returning to our PAF example, one could estimate PAF_AGG _for both smoking and birth weight by contrasting children who had neither of these risk factors, with those who have one or both of them. The result would be an entirely valid estimate of the total or net effect of both of these variables when examined simultaneously.

##### Adjusted Estimates

These examples address situations where one is interested in either the individual effect of a single predictor or risk factor, or where one is interested in the total or combined effect of several variables or risk factors examined simultaneously. Neither involves adjusting individual effects. However, as we have noted previously, researchers are also often interested in examining the effect of individual predictors or risk factors after statistically controlling for the effect of other variables. This might be due to an interest in statistically controlling for other potential confounding effects, or in order to control for the effect of earlier steps in a more complex process.

Returning to our R^2 ^analogy, in regression this can be done through a hierarchical multiple regression, where variables are entered sequentially in multiple steps, examining the change in R^2 ^at each step. For example, one might find R^2 ^= .30 for the first step (child sex only), then find a change R^2 ^equal to .10 when early childhood parenting is added in the second step (for a total R^2 ^= .40 after step 2), and finally obtain a change in R^2 ^equal to .05 when adolescent peer behavior is added in the third step (for a total R^2 ^= .45 after step 3). Note that the estimates of the change in R^2 ^do not separate direct and indirect effects associated with that variable. For example, the .10 change R^2 ^for early childhood parenting reflects both any direct effect it has on the outcome, as well as any indirect effect it may have through peer behavior.

The change-in- R^2^'s seen in each individual step sum to the total R^2 ^obtained in the final step, which is also identical to the total R^2 ^obtained by entering all three variables simultaneously in a single step. Combining the different approaches, one can estimate the total effect of each variable on its own (the simple r^2^'s), the total effect of all variables when examined together (the total R^2^), and the unique effect of individual variables controlling for one or more other variables (the change in R^2^'s). This approach is valuable in that it allows one to examine the relative process through which these variables influence the outcome, in conjunction to their individual net effects (r^2^'s) and the overall impact of all variables together (R^2^). Furthermore, if the effect of the predictors on the outcome is believed to be confounded by other variables, those variables can be entered in the first step in order to adjust for those possible confounding effects.

Unfortunately, this last step currently has no equivalent parallel in PAF analyses. What is needed is a procedure whereby multiple risk factors can be examined in two or more steps, with the PAF adjusted at each step for the effect of variables entered in earlier steps, and where these sequentially adjusted PAF estimates sum to the total PAF observed when all variables are examined simultaneously. This would provide the final, third parallel between R^2 ^as an indicator of the variance in an outcome associated with multiple predictors in multiple regression, and PAF as an indicator of the percentage of cases in a population associated with multiple risk factors. The procedure we are proposing accomplishes this task.

As described above, this procedure is designed to complement the information obtained by an unadjusted PAF and PAF_AGG_. Just like the simple r^2^, the simple, unadjusted PAF provides an estimate of the total or net effect of a risk factor. Similarly, just like the total R^2^, the PAF_AGG _provides an estimate of the net or total effect of a group of variables. However, this procedure provides additional, valuable information that supplements both of these. In situations where one has a causal-sequence of effects, such as maternal smoking leading to increased cases of babies born low birthweight, leading to increased numbers of children identified as having MMR, this can provide potentially interesting process information. Returning to our example, whereas the simple PAF for low birth weight indicates the total effect it has on cases of MMR, the adjusted PAF reflects how much of that effect is not driven by earlier processes (i.e., maternal smoking). This can provide valuable information for researchers interested in these developmental, longitudinal processes. Furthermore, if the effect of the risk factors on the outcome is believed to be confounded by other variables, those variables can be entered in the first step in order to adjust for those possible confounding effects.

### Previous techniques for partitioning a PAF sequentially

This approach differs from that proposed by Eide and Gefeller[15], who have also proposed a method of sequential PAF estimation. Their strategy was to add risk factors to a model one at a time and calculate the increase in the total PAF at each step. They do not suggest ordering variables based on a causal sequence, but instead propose that an optimal strategy would be to start with the risk factor having the largest individual PAF. The resulting estimate for each variable is referred to as a *sequential attributable fraction*. For example, they cite a previous study to illustrate how the PAF for smoking as a predictor of chronic cough was 41.2%, while the PAF of smoking and occupational dust exposure together was 51.2%. Therefore, the sequential PAF for smoking would be 41.2%, while the sequential PAF for occupational exposure would be 10.0%.

Eide and Gefeller[15] go on to propose that an *average PAF *can be calculated by estimating the mean sequential PAF for a given risk factor, based upon all possible orderings of variables in the model. In this way, if the mean sequential PAF is calculated for each variable in the model, the sum of the average PAFs equals the aggregate PAF obtained when all variables are examined together. While this addresses the issue of PAFs summing to more than 1.0, to quote Rowe and colleagues, estimates based on this approach "which assume complete elimination of one risk factor while the prevalence of the other risk factor remains static, do not represent realistic scenarios"[6] (p.246)

More importantly, while their approach is fairly straightforward, it does so by simply attributing the entire remaining portion of an aggregate PAF to subsequent risk factors. The result is that it does not allow for interactive effects (i.e., it assumes there is no interaction between risk factors as predictors of the outcome). As we will document in the final section of this paper, incorporating interactions into PAF estimates solves the seemingly paradoxical findings that have been noted by Wilcox[16]. By including possible interaction terms, our approach provides the correct answer to these paradoxical situations and directly addresses one of the key concerns with calculating adjusted PAF estimates.

### The proposed sequential partitioning strategy

In contrast to these other approaches, the sequential partitioning strategy we propose incorporates two key features. First, it recognizes that in multiple risk factor models one risk factor may in fact lead to a higher rate or prevalence of a subsequently occurring risk factor. This results in a risk factor having both a direct effect on the outcome, as well as indirect effects through increased rates of subsequent risk factors. Consider maternal smoking during pregnancy and low birth weight–both are related to MMR, but smoking has an indirect effect through increased rates of low birth weight[2]. As such, part of the effect of low birth weight is in fact due to the indirect effect of smoking and should be attributed to smoking, not to low birth weight. In such a model, low birth weight can be thought of as an intermediate or mediating risk factor. The need to address this is the very issue raised by Rowe and colleagues[6] (see the top example in Figure 1).

Second, while on a conceptual level, the issue of direct and indirect effects is important in determining the sequential order of variables, as well as in interpreting the resulting effects, the proposed strategy does not involve attempting to separate out or differentiate direct and indirect effects. This is an equally important point given concerns raised regarding difficulties in separating direct and indirect effects. Specifically, the PAF associated with the first risk factor reflects its total effect. It is a single value that reflects both any direct effect upon the outcome and any indirect effect through mediating variables. The adjusted PAF estimated for any subsequent risk factors removes the impact of all preceding variables on that risk factor, but still results in a single estimate that reflects both any direct effect of that variable upon the outcome and any indirect effect it may have that is mediated by variables appearing later in the process. Returning to the previous example, the PAF associated with smoking would reflect the total impact of smoking–both the effect it has directly upon MMR, and the indirect effect it has by increasing the number of children born low birth weight. The PAF associated with low birth weight would reflect that portion of the low birth weight effect that is unrelated to smoking. If a third risk factor was entered after low birth weight, the PAF for low birth weight would reflect any direct effect of low birth weight (controlling for smoking) and any indirect effect it has through that third risk factor (again, controlling for smoking). This also highlights the point that if the effect of the risk factors on the outcome is believed to be confounded by other variables, those variables can be entered in the first step in order to adjust for those possible confounding effects.

The proposed procedure begins with a PAF (PAF_AGG_) describing the total aggregated effect of all risk factors, and then partitions this PAF based upon the sequential order of the effects in the model. Similar to the stratified approach, the sum of the PAFs obtained is equal to the PAF that would be obtained by simply placing all individuals with one or more of the risk factors into a "risk" group, and then calculating a PAF for this aggregate indicator of "risk" relative to those with none of the risk factors. This is an important characteristic, in that it emphasizes the strategy is partitioning the total, net effect of all the risk factors in the model. In addition, similar to adjustment approaches, it addresses issues that arise from risk factors being correlated, as well as risk factors having interactive effects. Finally, based upon the sequence of effects, this new procedure adjusts the prevalence of risk factors, p_e_, not just the RR, at each step.

For simplicity and transparency, we focus on two risk factors entered in two steps; however, the process can readily be continued to include additional risk factors or potential confounds across 3 or more steps. We begin by describing the procedure for situations where two risk factors are related/correlated with each other, but do not interact. We then address the case where two risk factors are not related/correlated with each other, but do interact. We then address the situation where the two risk factors are related/correlated with each other and interact.

It should be noted that given analyses are conducted using risk ratios, we are specifically referring to an interaction in the risk-additivity sense[17], meaning that the expected RR for a person experiencing risk factor A and risk factor B (assuming no interaction) is equal to the RR for A plus the RR for B minus 1. This contrasts with the multiplicative interaction as would be seen in the product term of a logistic regression, where the expected odds ratio for a person experiencing both A and B (assuming no interaction) is equal to the odds ratio of A multiplied by the odds ratio of B (see [17] for a thorough discussion regarding these distinctions).

Finally, we should once more note that while we have referred to indirect effects as a basis for establishing a sequential model, this procedure does not attempt to differentiate direct and indirect effects. The partitioned PAF for any variable contains both any direct effect that variable has on the outcome, as well as any indirect effect it may have through subsequent variables in the model, after removing the effect of any earlier variables in the model. This is in the same manner that the change in R^2 ^for a variable entered in a multi-step hierarchical multiple regression reflects the net effect (both direct and indirect through any subsequent variables) of that variable, after removing the variance associated with any variables that had been entered on a previous step.

### Computational illustrative examples

#### No interaction among risk factors

**Figure 3:**
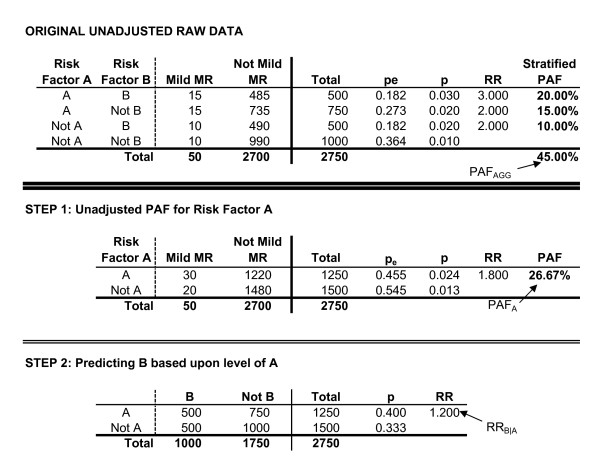
Two risk factors with no interaction.

**Figure 4:**
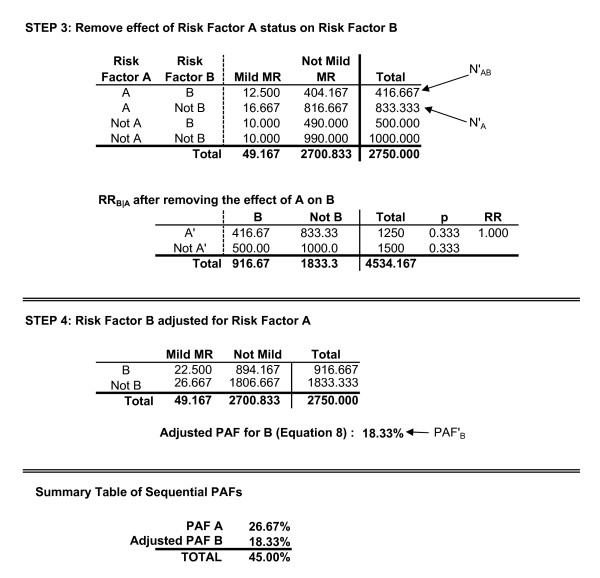
**Two risk factors with no interaction.** (Continued).

The first example involves two risk factors, A and B, where A is believed to lead to increased rates of B, and both are believed to result in elevated rates of MMR. A and B are related but have no interaction effect. For example, smoking may lead to higher rates of low birthweight, and low birthweight may lead to higher rates of MMR; but the effect of being born low birthweight may be identical for all children, regardless of whether or not their mother smoked during pregnancy. Data for this example are presented in Figures 3 and 4.

##### Step 1

The first step is to calculate an unadjusted PAF for risk factor A using the general PAF formula...

(3) $$PAF_{A}=\frac{P_{e}(RR-1)}{1+P_{e}(RR-1)}$$

where P_e _is the proportion of the population exposed to risk factor A. This is the total relationship between risk factor A and population rates of MMR. This includes both its direct effect that is unrelated to B, and the indirect effect it has through increased rates of B. For this example, the unadjusted PAF for A is equal to 26.67%.

##### Step 2

The next series of steps adjust the rate of risk factor B so as to remove the effect of A upon B. To do this, one first calculates RR_B|A_, which is the risk ratio for B based upon exposure to A. In other words, RR_B|A _treats the presence of risk factor B as the "outcome", and estimates the increased risk of B among individuals with risk factor A, relative to the risk of B among individuals without A. For this example, RR_B|A _is equal to 1.20, indicating the children who experience A are 1.20 times more likely to experience B, than are children who did not experience A.

##### Step 3

The next step involves creating an adjusted frequency table by adjusting frequencies among those individuals with risk factor A in order to remove any effect that A may have had in terms of increasing rates of risk factor B. In essence, this adjusted table reflects the predicted frequencies given no association between A and B. RR_B|A _quantifies the relationship between A and B, in that it indicates the increased probability that a person will experience B if they have also experienced A. Therefore, multiplying the number of individuals with both A and B by the inverse of RR_B|A_, while keeping constant both (1) the total number of individuals experiencing A, and (2) the probability of having the outcome of interest, removes any effect of A on rates of B. Mathematically, this process is described in equations 4 through 7, below.

Referring to Figure 4, the adjusted number of individuals experiencing both risk factor A and risk factor B (N'_AB_) is equal to

(4) $${N^{\prime }}_{AB}=N_{AB}RR_{B|A}^{-1}$$

where N_AB _is the original number of individuals with both A and B. By removing the effect of A upon B, the total number of individuals in this group would change; however, the probability of the outcome among these individual (p'_Case|AB_) is not affected and continues to be equal to .030, so that...

(5) *p'*_*Case*|*AB *_= *p*_*Case*|*AB*_

where p_Case|AB _is the unadjusted probability of the outcome among individuals with both A and B. As reflected in Figure 4, adjusting for the relationship between the two risk factors, the expected number of individuals with both A and B would be equal to 416.67.

Through the adjustment process, the total number of individuals exposed to A does not change (i.e., continues to equal 1250), consequently any change in the number of individuals experiencing both risk factors must be offset by a corresponding change in the number of individuals who experience A but not B (N'_A__). Mathematically, this is equal to...

(6) *N'*_*A_ *_= *N*_*AB *_+ *N*_*A_ *_- *N'*_*AB*_

where N_A_ _is the number of individuals in the original data with A who do not have B. In this example, N'_A_ _equals 833.333. As before, the probability of developing the outcome among these individuals does not change...

(7) *p'*_*Case*|*A_ *_= *p*_*Case*|*A_*_

where p'_Case|A_ _is the adjusted probability of the outcome among this group, and p_Case|A_ _is the probability of the outcome among this group in the original data (i.e., among individuals experiencing A but not B, both the adjusted and unadjusted probability of having MMR is equal to .020). The effect of this adjustment is to make the risk ratio for B based upon A equal to one.

##### Step 4

The adjusted frequency table is then aggregated based upon exposure/lack of exposure to risk factor B, allowing an adjusted PAF of B. However, Equation 3 is now inappropriate as it would not refer to the original number of cases. The adjusted PAF (PAF'_B_) is instead calculated based on an equivalent form of Equation 3 that has then been slightly modified in order to reflect the adjusted number of cases relative to the original, unadjusted number of cases...

(8) $$PA{F^{\prime }}_{B}=\frac{\left[{N^{\prime }}_{Case}-{p^{\prime }}_{Case|not\begin{array}{c} B \end{array}}\times N\right]}{N_{Case}}$$

Specifically, N_case _is the total number of cases of the outcome in the original table, N'_case _is the total number of cases in the adjusted table, p'_Case|Not B _is the probability of the outcome among those in the adjusted table without risk factor B, and N is the total number of individuals in the sample. In effect, the numerator estimates the reduction in the number of cases based on the adjusted data, which would be observed if those experiencing B had the same probability of the outcome as did those who did not experience B; while the denominator is the number of cases observed in the unadjusted data. The result is the proportion of cases in the original, unadjusted data related to the adjusted data for B. By making this estimate relative to the unadjusted number of cases, it can be combined with the previously estimate PAF for A, which was also relative to the unadjusted number of cases.

This results in an adjusted PAF for B equal to 18.33%. Given the adjustment process removes any relationship between A and B, the unadjusted PAF for risk factor A (PAF_A_) and the adjusted PAF for risk factor B (PAF'_B_) sum to the overall, aggregate PAF for A and B combined (PAF_AGG_) if there is no interaction.

### Interactive effects

#### Risk factors have an interaction effect, but are not related/uncorrelated with each other

**Figure 5:**
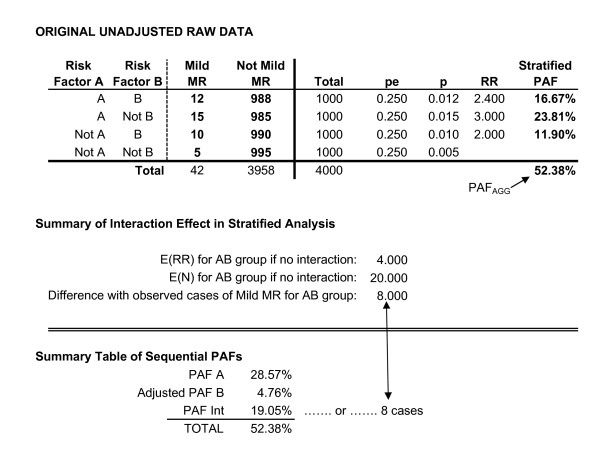
Two unrelated risk factors with an interaction.

These next two examples illustrate how sequential partitioning is applied to data in which the risk factors have an interaction effect. We will first consider an interaction in a stratified analysis with risk factors that are unrelated/uncorrelated with each other. For example, there may be no association between child gender and maternal smoking (i.e. child gender has no indirect effect on the outcome through maternal smoking); however, being both male and having a mother who smoked may result in greater risk than would be expected given the individual risks of being male and maternal smoking, alone. As noted previously, a stratified analysis would be acceptable given the risk factors are not related to each other. Figure 5 contains hypothetical data for which an interaction exists between two unrelated risk factors, A and B, as predictors of MMR. Assuming no interaction, the expected risk ratio among individuals with both risk factors is equal to...

(9) *E*(*RR*_*AB*_) = *RR*_*A_ *_+ *RR*_*_B *_- 1

where RR_AB _is the risk ratio for individuals with both risk factors, RR_A_ _is the risk ratio for individuals with A but not B, and RR__B _is the risk ratio for individuals with B but not A. For the data in Figure 5, the expected RR_AB _with no interaction would be 4.00, translating to 20 cases of MMR. The observed number of cases of MMR for individuals in this group was 12. The difference, 8 cases, reflects the interaction effect (see [17] for detail regarding estimation of interactive effects).

It was previously noted that with non-interacting risk factors, PAF_AGG _was equal to the sum of the unadjusted PAF for A and the adjusted PAF for B. However, in this interacting example, PAF_A _and the adjusted PAF_B _sum to 33.33%, which is 19.05% less than PAF_AGG_. This 19.05% corresponds to 8 of the 42 cases of MMR, which, given the risk factors are not related to each other, is also equal to the magnitude of the interaction effect obtained in the stratified analysis. Consequently, the interaction in a sequentially partitioned PAF (PAF_Inter_) is equal to...

(10) *PAF*_*Inter *_= *PAF*_*AGG *_- [*PAF*_*A *_+ *PAF'*_*B*_]

And expressed as a number of cases....

(11) *N*_*Inter *_= (*PAF*_*AGG *_- [*PAF*_*A *_+ *PAF'*_*B*_])* *N*_*Cases*_

where N_Cases _is the number of cases of the outcome (MMR) in the original sample.

#### Risk factors interact and are related/correlated with each other

**Figure 6:**
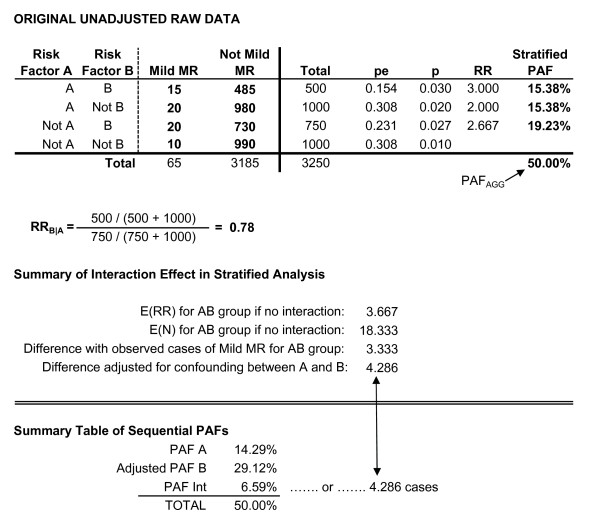
Two correlated risk factors with an interaction.

The final example considers the situation where there is an interaction involving risk factors that are related/correlated with each other. For example, (1) maternal smoking during pregnancy may lead to higher rates of babies born low birthweight, while low birthweight then leads to higher risk of MMR (i.e., smoking and birthweight are related) *and* (2) the effect of being born low birthweight on MMR may be different for those babies whose mothers also smoked, than is the effect of low birthweight for those babies whose mothers did not smoke (i.e., a smoking × birthweight interaction on MMR). Data for this example are presented in Figure 6. Applying the sequential partitioning strategy, PAF_A _is equal to 14.29%, the adjusted PAF_B _is equal to 29.12%, and the PAF for the interaction is equal to 6.59%. Applying Equation 11, the PAF for the interaction translates to 4.286 cases of the outcome.

In contrast, if we examine the same data using a stratified analysis, we would focus on the 500 individuals with both risk factors. Using Equation 9 and additional computation, the expected number of cases of MMR among individuals with both A and B is 18.33, while the observed number is 15, or a difference of 3.33. However, due to the fact that the risk factors are related/correlated, this value is biased and therefore does not equal the result obtained in the sequential approach. Nevertheless, the bias can be corrected by multiplying this result by the inverse of the RR for the occurrence of B given a person also experiences A (RR_B|A_^-1^). In other words...

(12) *N*_*INT*-*SEQ *_= *N*_*INT*-*START *_*RR*_*B*|*A*_^-1^

where N_INT-SEQ _is the number of cases of the outcome that were associated with the interaction using the sequential partitioning approach, and N_INT-STRAT _is the biased estimate of the number of cases of an outcome associated with the interaction when one inappropriately applies the stratified method. Applying this correction results in a value of 4.286 cases, which is the same as obtained through the sequential partitioning approach. Note that this correction does not make the stratified approach appropriate when risk factors are related/correlated. It is used in this instance simply to highlight a limitation of stratification in such instances, and to illustrate how the sequential approach provides a logical and easily understandable correction for this issue.

### Population shifts: sequential partitioning as a solution to an otherwise paradoxical effect

Finally, it is worth noting how the sequential partitioning procedure addresses issues resulting from population shifts in the frequencies of risk factors. Wilcox [16] described how in a study of the effects of birthweight and altitude upon infant mortality, it is possible for the birthweight frequency and mortality distribution to shift based upon altitude. In effect, the shapes of the curves do not change, but the optimal birthweight does.

As detailed by Wilcox [16], this can result in a number of seemingly paradoxical findings. For example, while increased altitude is associated with lower mean birthweight, and while lower birthweight is associated with increased mortality, altitude has no relationship with mortality. Furthermore, among infants born low birthweight, the mortality rate among high altitude births is less than the mortality rate among low altitude births. In contrast, among infants born with a high birthweight, the mortality rate among high altitude births is greater than that seen among low altitude births.

To illustrate how the sequential partitioning approach addresses these issues, an artificial data set was created reflecting a hypothetical relationship between altitude, birthweight, and mortality. Artificial data were used in order to ensure that the only effect associated with altitude was the shift in optimal values. This would allow the manner in which the partitioning approach addresses such shifts to be most evident. Specifically, two samples were created. The first, representing "low altitude" births had a mean birthweight of 3500 g, which was normally distributed with a standard deviation of 1000 g. A second sample, representing "high altitude" births had a mean birthweight of 3200 g and was also normally distributed with a standard deviation of 1000 g. Mortality rates per 1000 births was equal to

(13) $${\text{Mort}}_{1000}=e^{\left|\left({\text{W}}_{\text{POP}}-{\text{W}}_{X}\right)\right|/400}$$

**Figure 7:**
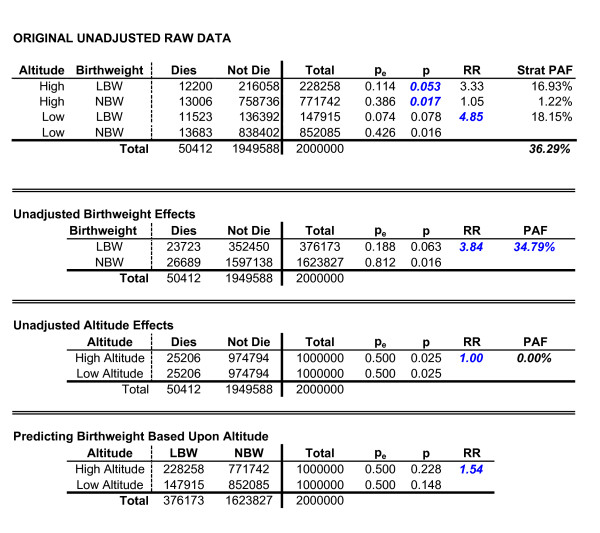
Hypothetical Example Involving Population Shifts-Unadjusted Estimates.

Where Mort_1000 _is the mortality rate per 1000 births, W_POP _is the mean birthweight in grams for a given population, and W_X _is a child's birthweight in grams. This resulted in a mortality rate of 1 per 1000 births at the mean population birthweight, and 518 per 1000 births two and a half standard deviations from the mean. Weights in each sample ranged from 2.5 standard deviations below the mean to 2.5 standard deviations above their corresponding mean, with each sample containing 1,000,000 births. Using a criterion for low birthweight as being less than 2500 grams, results are presented in Figure 7. For clarity, unless otherwise noted, all values referenced in the subsequent material are explicitly identified in Figure 7 with italics and bold blue font.

As expected, high altitude is related to low birthweight (RR = 1.54) and low birthweight is related to mortality (RR = 3.84); however, reflecting the paradox, altitude is unrelated to mortality (RR = 1.00). Furthermore, as expected, among high altitude births, the effect of low birthweight upon mortality (RR = .053/.017 = 3.17) is lower than the effect seen among low altitude births (RR = 4.85). For example, while not presented in Figure 7, the mortality rate among children born 1500 g was 70.1 per 1000 high altitude births and 148.4 per 1000 low altitude births. In contrast, the mortality rate among high altitude infants born high birthweight is in fact greater than the mortality rate among low altitude infants born high birthweight (90.0 per 1000 high altitude births > 5000 g, and only 42.5 per 1000 low altitude births > 5000 g). This exactly reflects the paradox resulting from population shifts that is noted by Wilcox. However, these seemingly paradoxical patterns disappear if one adjusts for altitude prior to examining the effect of birthweight [16].

**Figure 8:**
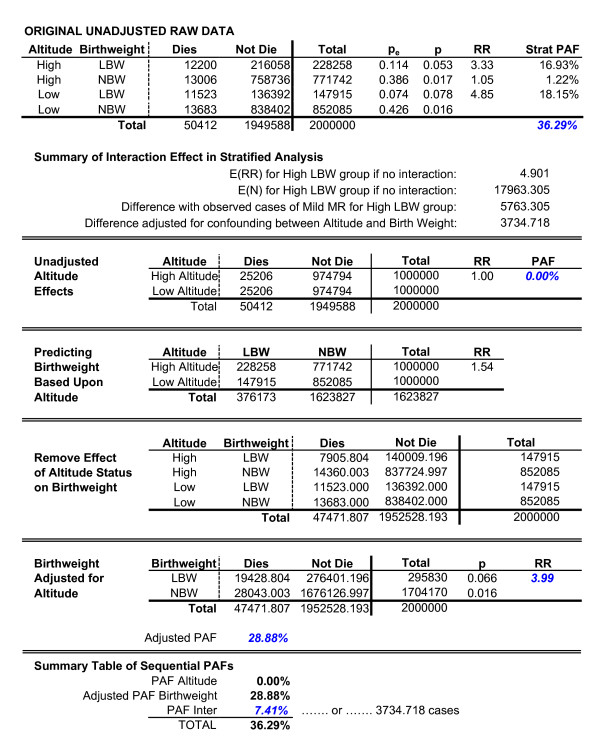
Hypothetical Example Involving Population Shifts–Adjustment Calculations.

Fortunately, the sequential partitioning technique incorporates just such a procedure, and furthermore, quantifies the degree to which a PAF may be impacted by this population shift. As presented in Figure 8, the overall PAF for altitude and birthweight is 36.29%. Based on the model that high altitude leads to lower birthweight, the sequential partitioning approach results in a PAF for altitude equal 0%. In other words, the conclusion would be that altitude has no relationship with mortality. This in fact corresponds to the data, and is also the conclusion Wilcox notes one should draw in situations where this type of population shift occurs [16].

Furthermore, using the sequential partitioning approach, the adjusted PAF for birthweight is equal to 28.88%, with an adjusted risk ratio of 3.99. In contrast, the unadjusted PAF for birthweight (as reported in Figure 7) is 34.79%, with an unadjusted risk ratio of 3.84. The difference between the adjusted values calculated using the partitioning strategy and the raw unadjusted values is also exactly what one would expect. Specifically, given the downward shift observed in the distribution for the high altitude birth and mortality curves, a portion of the high altitude births will inappropriately be classified as low birthweight, when in fact within their population, they are not low birthweight. This results in an unadjusted risk ratio lower than would be expected were one to include only the "true" cases of low birthweight, relative to each population. As this suggests, the unadjusted risk ratio is slightly smaller than the adjusted RR.

In contrast, the improper inclusion of population-specific normal birthweight infants in the low birthweight group results in an exaggerated PAF for birthweight. As would therefore be expected, the adjusted PAF for birthweight is in fact somewhat smaller than the unadjusted PAF for birthweight. Finally, given the only difference between low and high altitude births is the shift in the distributions, the interaction effect (PAF = 7.41%) quantifies the degree to which the aggregate PAF (36.29%) capitalizes on the definition of "low birthweight" being misapplied to a population where this shift has occurred.

It is worth noting that very different conclusions would be drawn using alternative strategies in which all risk factors are simultaneously adjusted for all other effects. For example, the unadjusted odds ratio for birthweight is 3.84, while the altitude-adjusted Mantel-Haenszel odds ratio for birthweight is 4.05. When calculating an adjusted PAF, Equation Two incorporates the larger, adjusted odds ratio, but does not consider the impact of altitude upon rates of low birthweight, and so p_e _is unchanged. Consequently, a constant p_e _and a larger odds ratio will increase the value for the PAF and lead to the conclusion that birthweight has a larger effect than it actually does. Similarly, the unadjusted odds ratio for altitude is 1.00, while the birthweight-adjusted Mantel-Haenszel odds ratio for altitude is .86. A constant p_e _and a smaller odds ratio will result in a smaller, in this case negative, PAF. One would therefore conclude that altitude is a protective factor–again counter to the correct finding that altitude has no effect.

## Summary

It should be noted that this procedure does not address or prove "causality". The issue of establishing and quantifying causality in epidemiological research is a topic of ongoing theoretical and philosophical debate [18-23]. Instead, this is a descriptive procedure providing a measure of the relative population-level effects of multiple risk factors based on a specific model that may or may not be true. As noted previously, the results will differ depending upon the specific order of effects indicated by a model. The question remains whether a proposed model is or is not plausible. Nevertheless, the sequential partitioning strategy proposed here provides a valuable alternative means for examining the population-level impact of multiple risk factors unavailable through other techniques that provide less clear or less meaningful values. The procedure allows one to partition the overall effect of multiple risk factors based upon the sequence of effects that exists among the variables. In essence, it allows researchers to incorporate into their models the effect that one risk factor may have in terms of increasing rates of other risk factors in the model. In addition, if the effect of a risk factor on the outcome is believed to be confounded by other variables, those variables can be entered in the first step in order to adjust for those possible confounding effects. Consequently, the technique provides a potentially valuable tool for researchers interested in multiple risk factor models. A Microsoft Excel file [Partitioning PAF Excel Tool.xls] containing an annotated worksheet for the calculations and procedures reported here is available online through the journal website 1.

## Competing interests

The authors declare that they have no competing interests.

## Authors' contributions

CM and ST contributed equally to the conceptualization and preparation of the manuscript. Both authors have read and approved the final manuscript.

## Supplementary Material

Additional file 1 Partitioning PAF Excel Tool. A Microsoft Excel sheet providing annotated partitioned PAF estimates and corresponding calculations. Click here for file
